# Biobran/MGN-3, an Arabinoxylan Rice Bran, Protects against Severe Acute Respiratory Syndrome Coronavirus 2 (SARS-CoV-2): An In Vitro and In Silico Study

**DOI:** 10.3390/nu15020453

**Published:** 2023-01-15

**Authors:** Mamdooh Ghoneum, Shaymaa Abdulmalek, Hewida H. Fadel

**Affiliations:** 1 Department of Surgery, Charles R. Drew University of Medicine and Science, 1731 E. 120th Street, Los Angeles, CA 90059, USA; 2 Department of Surgery, University of California Los Angeles, Los Angeles, CA 90095, USA; 3 Department of Biochemistry, Faculty of Science, Alexandria University, Alexandria 21511, Egypt; 4 Department of Medical Laboratory Technology, Faculty of Allied Medical Sciences, Pharos University in Alexandria, Alexandria 21648, Egypt

**Keywords:** Biobran, ACE2, antiviral, SARS-CoV-2, Vero

## Abstract

Severe Acute Respiratory Syndrome Coronavirus 2 (SARS-CoV-2), the causative agent of Coronavirus Disease 2019 (COVID-19), poses a serious global public health threat for which there is currently no satisfactory treatment. This study examines the efficacy of Biobran/MGN-3 against SARS-CoV-2. Biobran is an arabinoxylan rice bran that has been shown to significantly inhibit the related influenza virus in geriatric subjects. Here, Biobran’s anti-SARS-CoV-2 activity was assessed using MTT and plaque reduction assays, RT-PCR, ELISA techniques, and measurements of SARS-CoV-2-related gene expression and protein levels. For Vero E6 cells infected with SARS-CoV-2, Biobran reduced the viral load by 91.9% at a dose of 100 μg/mL, it reduced viral counts (PFU/mL) by 90.6% at 50 μg/mL, and it exhibited a significant selectivity index (EC_50_/IC_50_) of 22.5. In addition, Biobran at 10 μg/mL inhibited papain-like proteinase (PLpro) by 87% and ACE2 SARS-CoV-2 S-protein RBD by 90.5%, and it significantly suppressed SARS-CoV-2 gene expression, down-regulating E-gene and RdRp gene expression by 93% each at a dose of 50 μg/mL and inhibiting the E-protein by 91.3%. An in silico docking study was also performed to examine the protein–protein interaction (PPI) between SARS-CoV-2 RBD and DC-SIGN as well as between serine carboxypeptidase and papain-like protease PLpro. Serine carboxypeptidase, an active ingredient in Biobran, was found to interfere with the binding of SARS-CoV-2 to its receptor DC-SIGN on Vero cells, thus preventing the cell entry of SARS-CoV-2. In addition, it impairs the viral replication cycle by binding to PLpro. We conclude that Biobran possesses potent antiviral activity against SARS-CoV-2 in vitro and suggest that Biobran may be able to prevent SARS-CoV-2 infection. This warrants further investigation in clinical trials.

## 1. Introduction

Coronaviruses have emerged as significant global health threats. They had caused two earlier pandemics—the severe acute respiratory syndrome that appeared in China in 2003 [1] and the Middle East respiratory syndrome [2]—before causing the current Coronavirus Disease 2019 (COVID-19) pandemic that arose from Wuhan, Hubei Province, China. Bats are known to be the primary animal reservoir for coronaviruses, and the viruses have been able to mutate and adapt to infect humans, resulting in an animal-to-human species barrier jump [3]. The emergence of novel coronaviruses continually poses a serious global public health threat and carries the potential for causing major pandemic outbreaks in the naïve human population. Severe Acute Respiratory Syndrome Coronavirus 2 (SARS-CoV-2) is the coronavirus causing the disease COVID-19, a disease whose outbreak has resulted in over 239 million confirmed cases of infection and killed over 4.8 million people across the globe [4]. This novel virus has been globally transmitted to 228 countries and territories.

SARS-CoV-2 is thought to have been transmitted from a zoonotic source and spreads via contact and direct transmission. The disease manifests symptomatically with cough, myalgia, fever, and severe respiratory failure. Reverse transcriptase PCR is used to confirm diagnosis, and managing COVID-19 is mainly carried out via supportive therapy, along with mechanical ventilation in severe cases. Attempts to reduce the public spread of SARS-CoV-2 consist mainly of preventive strategies, community containment, and disease isolation. Vaccine developments for eliminating the virus from the host remain an ongoing challenge [5,6]. Inhibiting the entry of viruses into host cells and/or preventing viral replication may represent therapeutic approaches for SARS-CoV-2. Angiotensin-converting enzyme 2 (ACE2) enzyme is a key receptor for SARS-CoV-2 viral entry, and CD209L (also known as L-SIGN) and CD209 (also known as DC-SIGN) are members of the *C*-type lectin superfamily that are significantly expressed in human endothelial cells, i.e., in macrophages and dendritic cells of the lymph nodes, liver, and lung, and are recognized by viral glycoproteins of SARS-CoV-2 via their *C*-terminal carbohydrate domain [7]. After entry into host cells, SARS-CoV-2 replicates and spreads, taking advantage of compounds such as papain-like protease (PLpro) to process viral proteins and generate a functional replicase complex [8]. New compounds which disrupt the activity of ACE2 and/or PLpro may be effective at mitigating SARS-CoV-2′s detrimental actions.

26 COVID-19 vaccines have been reported by the World Health Organization (WHO) to have been developed and evaluated in phase III clinical trials [9]. Several of these vaccines have been shown to be highly effective against the original strain of COVID-19 and its variants, including BNT162b2, AZD1222, mRNA-1273, and Sputnik V. In phase III trials, these vaccines were well tolerated and the most effective vaccines (>90%) in the prevention of symptomatic cases. However, protection against infection was observed to decline at 6 months for BNT162b2 and AZD1222, and serious adverse effects and myocarditis have been reported in a few cases [10]. There is no satisfactory treatment for the disease. Thus, it is essential to investigate therapeutic products, including the application of therapeutic natural products that are nontoxic, affordable, and capable of exerting a strong anti-COVID-19 effect.

Different types of natural products and herbal components have been examined for their potential use against SARS-CoV-2 [11,12,13,14,15,16,17,18,19,20,21,22,23,24,25]. These include scutellarein, caffeic acid, myricetin, quercetin, saikosaponin B2, silvestrol, tryptanthrin, psoralidin, lectins such as griffithsin, isobavachalcone, and steroids [16,17]. In addition, other natural products have been reported to exert anti-COVID-19 activity, including flavonoids [18], polyphenols [19], *Agathis robusta* bark essential oil [20], and propolis, bee honey, and their components [21]. *S. japonica* stem extract was shown to significantly inhibit coronavirus CoNL63 attachment in mammalian cells, indicating that it could be effective against infection in its early stage [22]; *S. nigra* berry extract was shown to significantly reduce virus titers in Vero cells infected with infectious bronchitis virus (a pathogenic poultry coronavirus) [23]; *S. nigra* fruit and flower extracts were shown to inhibit SARS-CoV-2 RBD and ACE2 receptor binding [24]; and Resveratrol, a phenolic compound produced by a variety of spermatophytes such as peanuts, mulberry, and grapes, has been shown to inhibit SARS-CoV-2 replication in cultured Vero cells [25].

Biobran/MGN-3, an arabinoxylan rice bran, is another natural product that potentially holds great promise. Biobran supplementation has been shown to reduce the incidence of influenza-like illnesses (ILI) in elderly subjects [26,27]. It is an arabinoxylan extracted from rice bran treated with hydrolyzing enzymes from Shiitake mushrooms [28], and in addition to reducing ILI in elderly subjects, it has been characterized as an antiviral agent with the ability to exert anti-HIV activity in vitro [28] and significantly reduce the viral load in patients with chronic hepatitis C virus infection [29]. In addition, Biobran has shown potential as an anticancer agent. It exhibited antitumor activity in mice bearing a solid Ehrlich carcinoma tumor [30] and synergized with conventional therapies for the treatment of hepatocellular carcinoma in human patients [31]. Biobran’s antiviral and anticancer activity has been attributed to its ability to act as a potent biological response modifier (BRM). It is known to activate different arms of the immune system such as natural killer (NK) cells [30,32,33,34,35,36], increase human T and B cell mitogen response [32], activate dendritic cells [37,38,39], and enhance the phagocytic activity by macrophages [40]. Furthermore, Biobran has been shown to exhibit a psychoneuroimmune modulatory effect by enhancing health-related quality of life in healthy older adults [41] and in cancer patients [35,42].

Based on these characteristics, we hypothesized that Biobran would exert antiviral activity against SARS-CoV-2. No previous study has explored the therapeutic potential of Biobran against coronaviruses, nor the mechanisms by which its active ingredients might counteract a coronavirus such as SARS-CoV-2 by inhibiting viral entry into host cells and/or preventing viral replication. The results of the present in vitro study demonstrate that our hypothesis is correct, namely that Biobran has a very potent antiviral effect against SARS-CoV-2. Furthermore, the potential mechanisms behind Biobran’s anti-SARS-CoV-2 effect are demonstrated via an in silico study of the docking of Ferulic acid and serine carboxypeptidase. These compounds are present in crude rice bran and wheat bran, and many studies have shown that these bran product compounds play a key role in the products’ anti-oxidant, anti-inflammatory, and anti-cancer properties [43,44,45]. Specifically with respect to the potential anti-SARS-CoV-2 activity of Biobran, we studied the binding of Ferulic acid and serine carboxypeptidase to PLpro and DC-SIGN, bindings which would have the positive effect of interfering with SARS-CoV-2′s cell entry and replication capabilities.

## 2. Materials and Methods

### 2.1. Biobran

Biobran is a denatured hemicellulose that is extracted from rice bran by reacting rice bran hemicellulose with carbohydrate-hydrolyzing enzymes obtained from Shiitake mushrooms. Biobran’s main chemical structure consists of arabinoxylan with an arabinose polymer in its side chain and a xylose in its main chain [28]. Daiwa Pharmaceutical Co. Ltd. (Tokyo, Japan) kindly provided the Biobran for this study. The Biobran was prepared in saline (0.9% *w*/*v*), and Biobran solutions were freshly prepared each day.

### 2.2. Anti-SARS-CoV-2 Assays

#### 2.2.1. Protein Interactions

The Biobran was examined for its ability to inhibit papain-like proteinase, the spike protein receptor-binding domain, and ACE2 binding [46]. To achieve this aim, the protein–protein interaction (PPI) between SARS-CoV-2 RBD (PDB:7e5o) and DC-SIGN (PDB:6ghv) was examined to evaluate whether SARS-CoV-2 RBD can enter host cells by interacting with DC-SIGN. In addition, the PPI between serine carboxypeptidase (PDB:3sc2) and papain-like protease PLpro (PDB:7nfv) was examined to explore the efficacy of Biobran’s active ingredients in counteracting SARS-CoV-2 infectivity.

##### Papain-Like Proteinase (PLpro)

40 μL of 142 nM PLpro in buffer A (50 mM HEPES, pH 7.5; 0.1 mg/mL bovine serum albumin (BSA), and 5 mM Dithiothreitol (DTT)) was dispensed in a 96-well plate and then incubated with 100 μL of different concentrations of Biobran for 5 min. Reactions were triggered by adding the fluorogenic substrate Arg-Leu-Arg-Gly-Gly-AMC (Enzo Biochem, USA) (RLRGG-AMC, 10 μL of 250 μM) to buffer A, forcefully shaking for 30 s, and incubating for 6 min. Next, 10 μL acetic acid (0.5 M) was used to halt the reactions, the solution was shaken for 30 s, and the fluorescence emission intensity was measured (wavelength of excitation: 360 nm; wavelength of emission: 460 nm). This allowed the measurement of the inhibition percentage (%).

##### Spike Protein Receptor-Binding Domain (RBD)

SARS-CoV-2 RBD (Abcam, UK) (1 μg/mL) was incubated with Biobran at 37 °C for 2 h, and the mixture was added to a 96-well plate, incubated overnight at 4 °C, and then blocked with 2% fat-free milk in phosphate-buffered saline with Tween^®^ detergent (PBST) at 37 °C for 2 h. Diluted ACE2 protein (Abcam, UK) was added to the plates and incubated again at 37 °C for 2 h. After four washes, bound protein was detected using hACE2-specific goat antibody (0.5 μg/mL, R&D system, McKinley, MN, USA) that was incubated for 2 h at 37 °C, followed by the incubation of horseradish peroxidase (HRP) conjugated anti-goat IgG antibody (1:5000, Thermo Fisher Scientific, Dreieich, Germany) for 1 h at 37 °C. The reaction was visualized by adding the substrate 3,3′,5,5′-Tetramethylbenzidine (TMB) (Sigma, St. Louis, MO, USA) and stopped using H_2_SO_4_ (1 N). An ELISA plate reader (Tecan, San Jose, CA, USA) was used to measure absorbance at 450 nm and thereby estimate the change in RBD–ACE2 complex formation.

#### 2.2.2. In Vitro Antiviral Activity

##### Vero E6 Toxicity

Biobran was diluted with Dulbecco’s Modified Eagle’s Medium (DMEM). Stock solutions of Biobran were prepared in 10% DMSO in ddH_2_O. Vero E6 cells (ATCC, CCL-81) were used to test cytotoxic activity by using the 3-(4, 5-dimethylthiazol -2-yl)-2, 5-diphenyltetrazolium bromide (MTT) method [47], modified slightly. Briefly, cells were seeded into 96-well plates (100 μL/well; 3 × 10^5^ cells/mL density) and incubated at 37 °C for 24 h in 5% CO_2_. After 24 h, various concentrations of Biobran were used to treat the cells in triplicates, and these were incubated for 24 h. After discarding the supernatant, sterile phosphate buffer saline (PBS) was used to wash the cell monolayers three times. MTT solution (20 μL of 5 mg/mL stock solution) was added to each well and incubated for 4 h at 37 °C, and then the medium was aspirated. Formazan crystals from each well were dissolved with 200 μL of acidified isopropanol (0.04 M HCl in absolute isopropanol, 0.073 mL HCl in 50 mL isopropanol). Using a multi-well plate reader, the absorbance of formazan solutions was measured at a maximum wavelength of 540 nm with a reference wavelength of 620 nm. The cytotoxicity percentage compared with untreated cells was determined using the following equation:

(1) $$\%\;\mathrm{cytotoxicity}=\frac{\mathrm{absorbance}\;\mathrm{of}\;\mathrm{cells}\;\mathrm{without}\;\mathrm{treatment}\;-\;\mathrm{absorbance}\;\mathrm{of}\;\mathrm{cells}\;\mathrm{with}\;\mathrm{treatment}}{\mathrm{absorbance}\;\mathrm{of}\;\mathrm{cells}\;\mathrm{without}\;\mathrm{treatment}}\times 100$$

The concentration exhibiting 50% cytotoxicity (IC_50_) was calculated using a plot of percentage cytotoxicity versus sample concentration.

##### Anti-COVID-19 Activity (Plaque Reduction Assay) 

Anti-COVID-19 activity was assessed via plaque reduction assay following the method of [48]. Vero E6 cells (1 × 10^6^ cells/mL) were cultivated in a six-well plate at 37 °C for 24 h. SARS-CoV-2 was diluted to 1 × 10^4^ plaque-forming unit (PFU)/well, mixed with 100 μL of Biobran’s safe concentration, and incubated at 37 °C for 1 h before being added to the cells. Growth medium was removed from the cell culture plates and the cells were inoculated with Biobran (100 μL/well). One hour of contact was allowed for virus adsorption, following which 3 mL of DMEM, 2% agarose, and the Biobran and virus were added onto the cell monolayer. The plates were left to solidify and incubated at 37 °C until viral plaques formed (3 days). Formalin (10%) was added for 2 h. Next, 0.1% crystal violet in distilled water was used to stain plates. The control wells were wells in which untreated virus was incubated with Vero E6 cells. Finally, the plaques were counted and the percentage reduction in plaque formation (% reduction) in comparison with the control wells was recorded according to the following equation:

(2) $$\%\;\mathrm{reduction}=\frac{\mathrm{Viral}\;\mathrm{count}\;\left(\mathrm{untreated}\right)-\mathrm{Viral}\;\mathrm{count}\;\left(\mathrm{treated}\right)}{\mathrm{Viral}\;\mathrm{count}\;\left(\mathrm{untreated}\right)}\times 100$$

##### Antiviral Activity

Twelve-well tissue culture plates were used to seed Vero E6 cells in DMEM containing 7% fetal bovine serum (FBS), 2 mM L-glutamine, 1 mM sodium pyruvate, 17.85 mM sodium bicarbonate, 15 mM HEPES, and 0.8 mM geneticin. Cells were seeded with 5% CO_2_ for 24 h at 37 °C prior to infection with SARS-CoV-2 at an MOI of 0.1 in infection media (maintenance media but containing only 2% FBS) for 2 h. The inoculum-containing media were removed and replaced with 1 mL fresh media (2% FBS) containing different Biobran concentrations or DMSO alone (10%) and incubated for 3 days. The cell supernatant was gathered and rotated at 6000 rpm for 10 min to remove debris, and the supernatant was transferred to fresh collection tubes. Cell monolayers were gathered by scraping and resuspending in 1 mL fresh media (2% FBS). Both the suspended cells and the supernatant were deactivated to kill the virus particles using UV, and cell lysis was then carried out with several steps of freezing and sawing [49,50].

The Qiagen viral RNA-isolation kit (#52906) was used to extract RNA from 200 μL aliquots of sample cell suspension or supernatant. The commercial first strand cDNA synthesis kit (Thermo Scientific, Waltham, MA, USA) was then used to perform reverse transcription according to the manufacturer’s instructions [51].

BetaCoV RdRp gene and BetaCoV E-gene amplifications were conducted using the following primer and probe sets. For the BetaCoV RdRp gene, 1 μM Forward (5′- AAA TTC TAT GGT GGT TGG CAC AAC ATG TT-3′) and 1 μM Reverse (5′- TAG GCA TAG CTC TRT CAC AYT T-3′) primers and 0.2 μM probe (5′-FAM- TGG GTT GGG ATT ATC-MGBNFQ-3′) (Abcam, Cambridge, UK) were used. For the BetaCoV E-gene, 1 μM Forward (5′-ACA GGT ACG TTA ATA GTT AAT AGC GT -3′) and 1 μM Reverse (5′-ATA TTG CAG CAG TAC GCA CAC A-3′) primers and 0.2 μM probe (5′-FAM-ACA CTA GCC ATC CTT ACT GCG CTT CG- 286 NFQ-3′) (Abcam, Cambridge, UK) were used. Ct values were calculated, converted to an expression quantifying the reduction in treated samples relative to the control using the ∆Ct method (fold change in viral RNA equals 2^∆Ct^), and expressed as a percentage of the DMSO sample alone.

The protein level of the envelope and spike proteins were analyzed with the ELISA technique using rabbit SARS-CoV-2 spike polyclonal antibody and SARS-CoV-2 envelope antibody (Abcam, Cambridge, UK), respectively. First, 100 μL of envelope and spike proteins (100 μg) were added to the well plate and incubated at room temperature for 2 h, and then at 4 °C overnight. The solution was removed, 200 μL of BSA was added, and then the well plate was incubated at 37 °C for 1 h. PBS was used to wash three times, and then the rabbit SARS-CoV-2 envelope or spike polyclonal antibodies were added to the wells and incubated at room temperature for 1 h. PBS was again used to wash three times, and then HRP conjugated anti-rabbit IgG antibody was added to the wells and incubated at room temperature for 1 h. The reaction was visualized by adding chromogenic substrate (TMB) and stopped using H_2_SO_4_ (1 N). An ELISA plate reader (Tecan, San Jose, CA) was used to measure the absorbance at 450 nm [52].

### 2.3. Docking

#### 2.3.1. Drug Docking

ChemSketch was used to draw the drug Ferulic, Avogadro energy was used for drug optimization, Spdbv was used for energy optimization of the proteins, Discovery Studio 2021 was used to visualize the ligand-receptor, and iGemodock was used for drug docking [53].

#### 2.3.2. Molecular Docking

ClusPro 2.0 was used to examine the protein–protein interaction (PPI) between SARS-CoV-2 RBD (PDB:7e5o) and DC-SIGN (PDB:6ghv) as well as the PPI between serine carboxypeptidase (PDB:3sc2) and papain-like protease PLpro (PDB:7nfv). The data were visualized and analyzed with the PyMol and Prodigy programs [54].

### 2.4. Statistical Analysis

Data were analyzed using one- and two-way analysis of variance (ANOVA) with the Sidak–Holm test, two-tailed unpaired Student’s *t*-test, and multiple *t*-test. *p* < 0.05 was used to determine statistical significance. GraphPad Prism 7.0 (San Diego, CA, USA) was used as the statistical software.

## 3. Results

### 3.1. The Binding/Inhibitory Effect of Biobran on Papain-Like Proteinase (PLpro), the Spike Protein Receptor-Binding Domain, and ACE2

The results depicted in Table 1 show that Biobran exerts a potent inhibitory effect against PLpro. Treatment with Biobran at a concentration of 10 μg/mL inhibited PLpro by 87%. Table 1 also shows that Biobran exerts a potent inhibitory effect against ACE2 SARS-CoV-2 S-protein RBD in a dose-dependent manner, with inhibition of 90.5% and 2.32% at 10 μg/mL and 0.625 μg/mL respectively.

### 3.2. Biobran Reduces Viral Load in Vero Cells Infected with SARS-CoV-2

Treatment with Biobran caused significant reduction in the viral load in Vero cells infected with SARS-CoV-2 (Table 2). A wide range of Biobran concentrations (3.1–100 μg/mL) were used to establish the dose response–relationship of its efficacy against SARS-CoV-2. Biobran exerts its effect in a dose-dependent manner. There was a 91.9% viral reduction in Vero cells post exposure to Biobran at a dose of 100 μg/mL, and a 16.1% viral reduction at a concentration of 3.125 μg/mL.

### 3.3. Vero E6 Toxicity and Plaque Reduction Assay

The antiviral activity of Biobran determined using Vero E6 toxicity and plaque reduction assays against SARS-CoV-2 is shown in Table 3. Compared with the initial viral counts, a significant reduction in viral counts (PFU/mL) following exposure to Biobran was observed. The toxic effect was dose dependent, with an inhibition of 90.6% at 50 μg/mL and 27% at 6.25 μg/mL.

### 3.4. IC_50_ and EC_50_ of Biobran

In order to evaluate the antiviral activity of Biobran, we examined the 50% inhibitory concentration (IC_50_) and the half maximal response (EC_50_). The results in Table 4 show that the Biobran Vero E6 IC_50_ = 15.36 μg/mL and the anti-COVID-19 EC_50_ = 3.46 μg/mL. These results indicate that Biobran exerts significant antiviral activity against SARS-CoV-2 with a high selectivity index of 22.5 for antiviral activity relative to cellular toxicity.

### 3.5. Biobran Suppresses SARS-CoV-2 Gene Expression and Protein Levels

The results in Table 5 show that treatment with Biobran causes significant suppression of SARS-CoV-2 gene expression for the E-gene and RdRp gene. Treatment with Biobran at 50 μg/mL, 25 μg/mL, 12.5 μg/mL, and 6.25 μg/mL caused down-regulation of the E-gene by 92.7%, 73.4%, 56.0%, and 21%, respectively. Similarly, treatment with Biobran resulted in the significant down regulation of RdRp by 93%, 81%, 64%, and 32%, respectively. In addition, the E-protein was significantly inhibited by 91.3%, 75.3%, 49.6%, and 19.4% post exposure to Biobran.

### 3.6. SARS-CoV-2 Docking with DC-SIGN

To confirm the specificity of the inhibitory effect of Biobran on SARS-CoV-2 in cultured Vero cells, we performed an in silico simulation and modeling of the structure of Biobran and the structures of the key nodes in this pathway.

**Figure 1:**
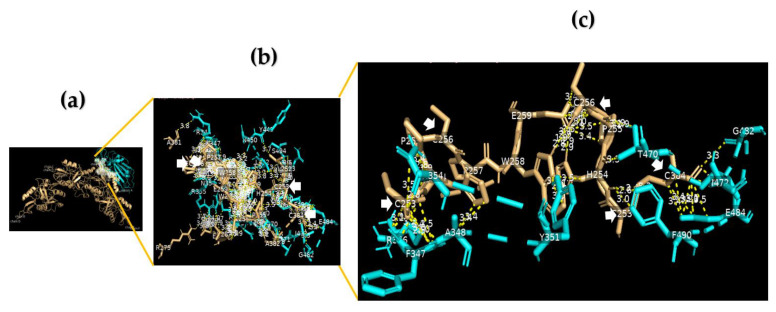
The RBD domain of the SARS-CoV-2 spike protein binds with DC-SIGN. (**a**) A 3D image for position (model 3) showing the molecular docking of the RBD domain of the SARS-CoV-2 spike protein (chain A) (cyan) to DC-SIGN CRD (light orange); (**b**) a 3D image showing protein–protein interaction (PPI) via binding of R346 F347 A348 S349 V350 Y351 A352 N354 R355 Y449 N450 L452 E465 R466 D467 I468 T470 E471 I472 G482 E484 F490 L492 Q493 S494 of chain A of the RBD domain of the SARS-CoV-2 spike protein (cyan) to C253 H254 P255 C256 P257 W258 E259 A381 of chain B and L252 C253 H254 P255 C256 W258 R275 E286 V287 A382 C384 of chain E of DC-SIGN CRD (light orange), using a cut-off of 4.0 Å; (**c**) a 3D image showing the binding sites between R346 F347 A348 N354 Y351 T470 G482 E484 F490 of the RBD domain of the SARS-CoV-2 spike protein (cyan) and C253 C256 of chain B and C253 C256 C384 of chain E of DC-SIGN CRD (light orange) using a cut-off of 3.5 Å.

Using a cut-off of 4.0 Å, we found that the RBD domain of the SARS-CoV-2 spike protein to DC-SIGN showed a protein–protein interaction (PPI) via the binding of V382 S383 T385 L387 N388 F392 F515 E516 L517 of chain A to L252 H254 P255 C256 P257 W258 of chain D. In addition, we found a PPI between E340 R346 A347 F348 N354 (chain A) of the SARS-CoV-2 RBD and E259 (chain E) and H254 and C384 (chain C) of DC-SIGN. Furthermore, G381 V382 S383 P384 L387 N388 F392 T430 L517 A522 of the SARS-CoV-2 RBD can bind DC-SIGN at many critical residues, including L252 C253 H254 P255 P257 W258 E259 V287 A382 S383 C384 of chain D. Moreover, we identified multiple bonds between R346 F347 A348 S349 V350 Y351 A352 N354 R355 Y449 N450 L452 E465 R466 D467 I468 T470 E471 I472 G482 E484 F490 L492 Q493 S494 of the SARS-CoV-2 RBD and C253 H254 P255 C256 P257 W258 E259 A381 of chain B and L252 C253 H254 P255 C256 W258 R275 E286 V287 A382 C384 of chain E of DC-SIGN (Figure 1, Figures S1–S4).

### 3.7. Drug Docking of DC-SIGN

**Figure 2:**
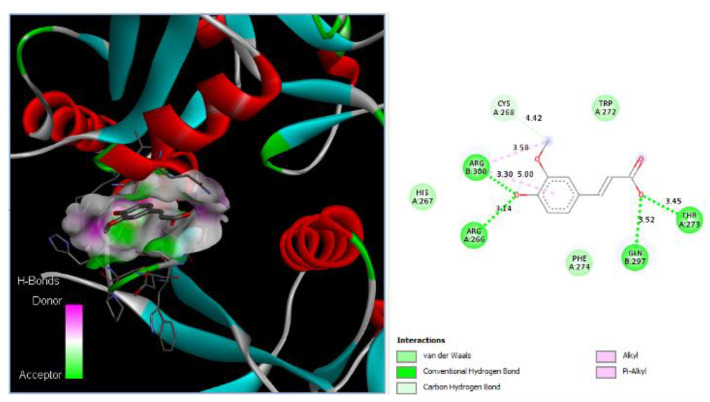
Ferulic acid binds to DC-SIGN: 3D image of Ferulic showing the hydrogen donor and acceptor (left) and 2D image showing the types of bonds and distances (right) between Ferulic and DC-SIGN where Ferulic can bind to Arg266, Thr273, Gln297, and Arg300 of DC-SIGN via hydrogen bonds with distances of 3.14 Å, 3.45 Å, 3.52 Å, and 3.30 Å, respectively. In addition, Ferulic can bind to Cys268 with carbon HBs with a distance of 4.42 Å, and Ferulic can bind to Arg300 via 2 alkyl bonds with distances of 3.58 Å and 5.00 Å.

Ferulic acid has been found to bind to proteins in DC-SIGN. Figure 2 shows that Ferulic can bind to Arg266, Thr273, Gln297, and Arg300 with hydrogen bonds with distances of 3.14 Å, 3.45 Å, 3.52 Å, and 3.30 Å, respectively. Additionally, Ferulic can bind to Cys268 with carbon HBs with a distance of 4.42 Å, and it can bind to Arg300 via 2 alkyl bonds with distances of 3.58 Å and 5.00 Å.

### 3.8. Molecular Docking of DC-SIGN

**Figure 3:**
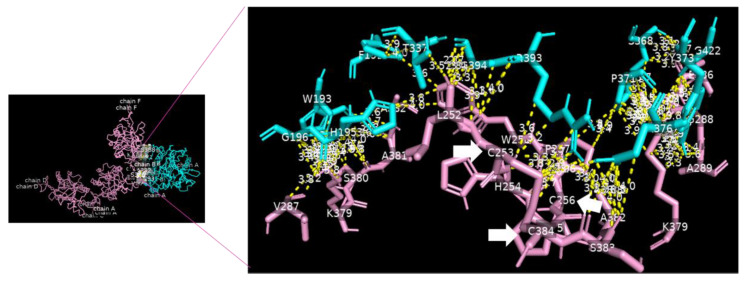
Serine carboxypeptidase binding to DC-SIGN: 3D image showing the interface between the whole structure of serine carboxypeptidase (cyan) and DC-SIGN (pink) (left side), with multiple binding sites between F191 W193 H195 G196 T337 S368 P371 Y373 I376 R393 G422 of serine carboxypeptidase (cyan) binding to L252 C253 H254 C256 W259 V287 G288 A289 K379 S380 A381 A382 S383 and C384 of DC-SIGN (pink) (right side) (cut-off: 4.0 Å).

**Figure 4:**
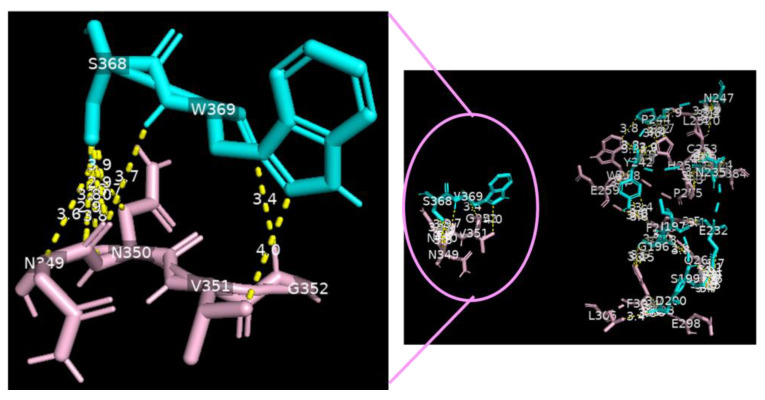
Serine carboxypeptidase binding to DC-SIGN: 3D image showing multiple binding sites between serine carboxypeptidase (cyan) and DC-SIGN (pink), including I197 S199 D200 E232 N235 Y242 P244 N247 E259 S368 W369 of serine carboxypeptidase (cyan) binding to L252 C253 W258 E259 E298 F302 L305 N349 N350 V351 G352 of DC-SIGN (pink) (right side) and a focus on binding between S368 W369 of serine carboxypeptidase (cyan) and N349 N350 V351 G352 of DC-SIGN (pink) (left side) (cut-off: 4.0 Å).

Analysis of the data collected from the molecular docking showed that serine carboxypeptidase can dock DC-SIGN with a binding affinity (ΔG) equal to −11.8 kcal mol^−1^ and a dissociation constant (K_d_) equal to 2.3 × 10^−9^ M, RMSD = 7.27 Å, and TM-score = 0.26528 if normalized by a length of 3sc2, or TM-score = 0.16107 if normalized by a length of 6ghv. Using a cut-off of 4.0 Å, we detected multiple binding sites between serine carboxypeptidase and DC-SIGN. As shown in Figure 3 and Figure 4, N294 N250 V251 G252 of serine carboxypeptidase can bind to C253 C265 and C384 of DC-SIGN, and I197 S199 D200 E232 N235 Y242 P244 N247 E259 S368 W369 of serine carboxypeptidase can bind to L252 C253 W258 E259 E298 F302 L305 N349 N350 V351G352 of DC-SIGN.

### 3.9. Drug Docking of PLpro

**Figure 5:**
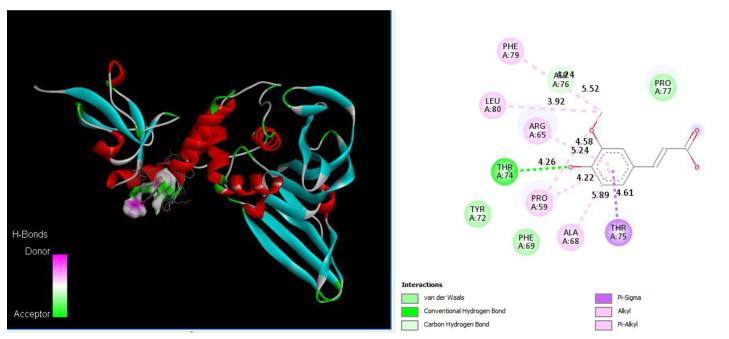
Ferulic acid docking PLpro by binding to Thr74 via a hydrogen bond (4.26 Å), binding to Asp76 via a carbon HB (3.42 Å), binding to Pro59 (4.22 Å, 4.58 Å), Arg65 (5.24 Å), Ala68 (5.89 Å), and Phe79 (5.52 Å), and Leu80 (3.92 Å) via alkyl bonds, and binding to Thr75 via a pi-sigma bond (4.61 Å). A 3D image showing hydrogen donor and acceptor (left) and a 2D image showing types of bonds and distances (right).

As shown in Figure 5, Ferulic acid binds to Thr74 via a hydrogen bond with a distance 4.26 Å, binds to Asp76 via a carbon HB with a distance 5.52 Å, and binds to Pro59 (4.22 Å, 5.24 Å), Arg65 (4.58 Å), Ala68 (5.89 Å), Phe79 (4.04 Å), and Leu80 (3.92 Å) via alkyl bonds. We also found that Chain B1 (N297 T299), Chain B2 (S303), and Chain B3 (D303 T304 N306 H308) of serine carboxypeptidase (PDB: 3sc2) can bind with V188 K190 K218 G219 Q221 Q229 E252 K315 of PLpro in SARS-CoV-2.

### 3.10. Molecular Docking of PLpro

**Figure 6:**
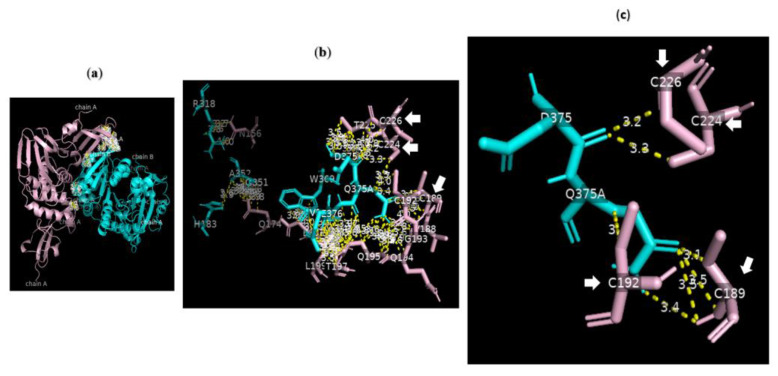
Serine carboxypeptidase can bind to PLpro: 3D images showing (**a**) the PPI between the whole structure of serine carboxypeptidase (cyan) and that of PLpro (pink); (**b**) the key residues in the interface between H183, R318, G351 A352 W369 D375 Q375A E376 V377 of serine carboxypeptidase (cyan) and N156 Q174 V188 C189 C192 G193 Q194 Q195 T197 L199 E203 C224 T225 C226 of PLpro (pink) in SARS-CoV-2 using a cut-off of 4.0 Å; and (**c**) the binding sites between D375 of chain B8 of serine carboxypeptidase and C224 and C226 of PLpro as well as those between Q375 of chain B9 of serine carboxypeptidase and C189 and C192 of PLpro using a cut-off of 3.5 Å.

**Figure 7:**
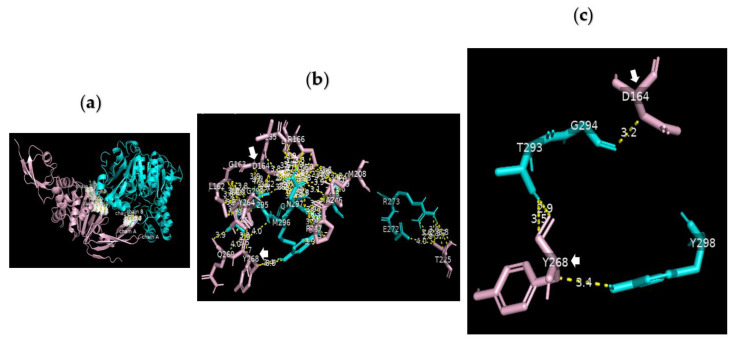
Serine carboxypeptidase can bind to PLpro: 3D images showing (**a**) the PPI between the whole structure of serine carboxypeptidase (cyan) and that of PLpro (pink); (**b**) the interface between E272 R273 Q285 N291 T293 G294 A295 M296 N297 Y298 T299 of serine carboxypeptidase (cyan) and L162 G163 D164 V165 R166 E167 M208 T225 A246 P247 Y264 Y268 Q269 Y273 of PLpro (pink) in SARS-CoV-2 using a cut-off of 4.0 Å; and (**c**) the binding sites between T293 and Y298 of serine carboxypeptidase and the key residues of PLpro (D164 and Y268) using a cut-off of 3.5 Å.

Analysis of the data collected from the molecular docking showed that serine carboxypeptidase can dock PLpro with a binding affinity (ΔG) equal to −7.8 kcal mol^−1^ and a dissociation constant (K_d_) equal to 2.0 × 10^−6^ M, RMSD = 6.73 Å, and TM-score = 0.092 if normalized by a length of 7nfv, or TM-score = 0.21743 if normalized by a length of 3sc2 (Figure 6 and Figure 7). The data showed that H183, R318, G351 A352 W369 D375 Q375A E376 V377 of serine carboxypeptidase can bind to multiple residues including N156 Q174 V188 C189 C192 G193 Q194 Q195 T197 L199 E203 C224 T225 C226 of PLpro in SARS-CoV-2. Additionally, E272 R273 Q285 N291 T293 G294 A295 M296 N297 Y298 T299 of serine carboxypeptidase can bind to L162 G163 D164 V165 R166 E167 M208 T225 A246 P247 Y264 Y268 Q269 Y273 of PLpro in SARS-CoV-2.

## 4. Discussion

The COVID-19 pandemic has been ongoing for almost three years, yet there remains an urgent need for new therapies that can curtail the spread of the disease and reduce mortality. The present study examined the anti-COVID-19 potential of Biobran/MGN-3 and the mechanisms underlying its effects. The results showed that Biobran is a potent anti-COVID-19 agent: it significantly inhibits ACE2–SARS-CoV-2 S-protein RBD binding, it significantly suppresses SARS-CoV-2 gene expression and protein levels, and it has a significant cytotoxic effect on Vero E6 cells with a high selectivity index.

For Vero cells infected with SARS-CoV-2, Biobran reduced the viral load by 91.9% at a dose of 100 μg/mL. This highly significant viral reduction is in accordance with other recent studies. Whole aqueous extract of Adhatoda Vasica reduced the viral load in Vero cells infected with SARS-CoV-2 [55], the combination of hydroxychloroquine and azithromycin shortened the duration of SARS-CoV-2 viral load in COVID-19 patients [56], and the oral disinfectant povidone-iodine (PVP-I) inhibited SARS-CoV-2 in vitro [57]. A wide range of Biobran concentrations (3.1–100 μg/mL) were studied here to establish the dose–response relationship of its efficacy against SARS-CoV-2. Biobran caused a 91.9% viral reduction in Vero cells at a dose of 100 μg/mL and a 16.1% viral reduction at a concentration of 3.125 μg/mL.

These data are also consistent with Biobran’s effect on SARS-CoV-2 replication, as analyzed with RT-PCR assays. Biobran treatment inhibited SARS-CoV-2 replication with an EC_50_ (half-maximal effective concentration) of 3.46 μg/mL and an IC_50_ (half-maximal inhibitory concentration) against Vero E6 of 15.36 μg/mL, resulting in a high selectivity index (EC_50_/IC_50_) of 22.5. This ratio quantifies antiviral activity relative to cellular toxicity. Recent studies have reported that drugs with high SI values (>10) could potentially be used as antiviral drugs, and, theoretically, the more elevated the SI ratio, the more efficient and safer a drug is [20,58].

Biobran strongly inhibited PLpro activity and downregulated the expression of RdRp, resulting in the downregulating of the viral structural protein and the preventing of viral replication. Treatment with Biobran at a concentration of 10 μg/mL caused an 87% inhibition of PLpro. Recent work has studied several SARS-CoV-2 PLpro inhibitors. For example, a topical antiseptic Acriflavine solution has been shown to inhibit PLpro and have antiviral effects [59], cysteine reactive Ebselen and Disulfiram compounds have been identified as SARS-CoV and MERS-CoV PLpro inhibitors [60], and several drugs for Hepatitis C virus (HCV) have shown inhibitory effects against SARS-CoV-2 PLpro. The latter is based on the observation that the substrate binding cleft and active site of the SARS-CoV-2 Mpro are structurally similar to the active site of the HCV NS3/4A protease [61,62]. HCV protease inhibitors show differential potency based on the detection of SARS-CoV-2 spike protein in Vero E6 cells [63].

Furthermore, Biobran suppressed SARS-CoV-2 gene expression and protein levels. Treatment with Biobran at different doses (50 μg/mL, 25 μg/mL, 12.5 μg/mL, and 6.25 μg/mL) caused significant E-gene down regulation (92.7%, 73.4%, 56.0%, and 21%, respectively) and E-protein inhibition (91.3%, 75.3%, 49.6%, and 19.4%, respectively). These results are in accordance with other studies on the anti-SARS-CoV-2 activity of plant extracts. For example, *S. nigra* berries and flowers have been shown to exert inhibitory activity against ACE2 SARS-CoV-2 RBD binding in vitro [24]; *S. nigra* berry extract can reduce virus titers in Vero cells infected with infectious bronchitis virus (a pathogenic poultry coronavirus) [23]; *Cannabis sativa* extract has been found to decrease ACE2 protein levels [64]; and *Agathis robusta* bark essential oil has shown effectiveness against COVID-19 [20].

ACE2–SARS-CoV-2 RBD binding is key to viral entry into host cells, and its inhibition may be a potent therapeutic approach for SARS-CoV-2. The ACE2 enzyme has been reported to be the main receptor for SARS-CoV-2 viral entry [65]. Complex formation between the host cell receptor and viral particles is mediated by the receptor binding domain (RBD) of the viral spike proteins and the extracellular peptidase domain of the ACE2 receptor [66]. In this study, Biobran demonstrated a potent inhibitory capacity against ACE2–SARS-CoV-2 S-protein RBD binding in a dose-dependent manner, suggesting that Biobran can interfere with the entry of SARS-CoV-2 into host cells.

To investigate the binding further, the present in vitro study was complemented with in silico research in which it was found that Ferulic acid and serine carboxypeptidase, both of which are active ingredients of Biobran, can bind to DC-SIGN and PLpro at critical residues, thus impairing their actions. Our docking study collectively showed that cysteine residues in DC-SIGN are the main target of the SARS-CoV-2 RBD protein, and that Ferulic can bind to Arg266, Thr273, Gln297 and Arg300 Cys268 of DC-SIGN. DC-SIGN is a *C*-type lectin-like molecule, and a previous study has shown that cysteine residues are crucial in interactions with viral glycoproteins at the binding sites of *C*-type lectin-like molecules [67]. We found that among the multiple binding sites, S368 W369 of serine carboxypeptidase can bind multiple residues in DC-SIGN, especially critical peptides that extend from N349 N350 V351 G352, which are crucial residues in the binding site. Thus, serine carboxypeptidase can bind to and block the binding site of DC-SIGN, subsequently interfering with the ability of SARS-CoV-2 to enter cells.

Furthermore, Ferulic acid can dock PLpro in SARS-CoV-2 by binding to Thr74, Asp76, Pro59, Arg65, Ala68, Phe79 and by binding to Leu80 via alkyl bonds. For serine carboxypeptidase, we found that N297 T299 of Chain B1, S303 of Chain B1, and D303 T304 N306 H308 of Chain B3 can bind with V188 K190 K218 G219 Q221 Q229 E252 K315 of PLpro in SARS-CoV-2. We also detected multiple binding sites between serine carboxypeptidase and the three catalytic zinc finger domains (finger, palm, and thumb) of PLpro in SARS-CoV-2. Of these, we found a bond between C192 and C226 which is among the four essential cysteine residues (C189, C192, C224, and C226) that form a zinc binding site of the finger domain of PLpro in SARS-CoV-2. Thus, Ferulic acid and serine carboxypeptidase can hinder the replication cycle of SARS-CoV-2 by binding to PLpro, subsequently impairing the assembly of SARS-CoV-2 proteins and subgenomic RNA. We found that Asp375 and Gln375 (chain B8 and chain B9, respectively) of serine carboxypeptidase chelated the four essential cysteine residues (Cys224, 226, 189, and 192) that are critical sites for zinc binding in PLpro. A previous study indicated that zinc binding is essential for the structural integrity and protease activity of PLpro [68]. We also found that T293 G294 Y298 of serine carboxypeptidase can bind to D164 and Y268 of PLpro in SARS-CoV-2 (D164 and Y268 are essential for the maintenance of PLpro) [8]. It is of interest to note that a recent study indicated that the binding site sequence of SARS-CoV-2′s PLpro is 100% identitical to that in SARS-CoV [69]. This indicates that Biobran could be an efficient inhibitor across the entire range of the SARS-CoV family.

Biobran’s effect against a range of coronaviruses was also indicated by our previous study in which we reported that Biobran supplementation significantly reduces the incidence of influenza-like illnesses (ILI) in elderly subjects [26]. That study showed that Biobran significantly upregulated the expression levels of retinoic-acid inducible gene I (RIG-I) and melanoma differentiation-associated protein 5 (MDA-5), as well as downstream ISG15 and MX1 in the human pulmonary epithelial BEAS-2B cell lines. The induction of RIG-1 and MDA-5 post exposure to Biobran has significant implications, since recent studies have reported the crucial roles of RIG-1 and MDA-5 in sensing and mounting effective antiviral responses against SARS-CoV-2 [70,71]. In addition, ISG-ylation of the caspase activation and recruitment domains of MDA-5 promotes its oligomerization and thereby triggers the activation of innate immunity against a range of viruses, including coronaviruses [72].

The current study is limited in its exploration of Biobran’s active ingredients. Further study is required to examine the mechanisms of additional active ingredients that may be involved in Biobran’s protection against SARS-CoV-2. The study is also limited in scope to in vitro measurements. The applicability of the current observations to live subjects must be studied to draw conclusions about the generalizability of the current results. Nevertheless, Biobran holds great promise, and it is a truly unique biological response modifier. Biobran does not exhibit hyporesponsiveness [35], making it a unique and attractive agent for long-term preventive and/or therapeutic purposes. Biobran has also been shown to be a completely safe and nontoxic agent, its biosafety has been demonstrated in many studies [35,73,74], it has been shown to reduce chemotoxic effects in animals [75,76] and to improve quality of life parameters in cancer patients [42,77], and it has not shown any adverse side effects in humans or animals after long periods of treatment [35,78,79].

## 5. Conclusions

Vero cells cultured with SARS-CoV-2 were used to study Biobran’s anti-COVID-19 effect in vitro. Biobran significantly reduced the viral load and viral count in infected Vero cells with a high selectivity index, it strongly inhibited viral entry by interacting with the ACE2 enzyme on the cell surface and inhibiting spike protein interaction, and it prevented viral replication by inhibiting PLpro activity and downregulating the expression of RdRp. Computational modeling further showed that serine carboxypeptidase, an active ingredient in Biobran, interferes with the binding of SARS-CoV-2 to its receptor DC-SIGN on Vero cells, thus preventing the cell entry of SARS-CoV-2. In addition, it binds to PLpro, thus impairing the viral replication cycle. Biobran may be a very useful and safe product for preventing and treating COVID-19 as an adjunct therapy, and further validations of its anti-SARS-CoV-2 applications are warranted.

## Figures and Tables

**Table 1:** The inhibitory/binding effect of Biobran on papain-like proteinase, the spike protein receptor-binding domain, and ACE2.

Biobran Concentration (μg/mL)	PLpro (Inhibition %)	Inhibition of Spike Protein RBD Complex Formation (%)
10	87.00 ± 0.42	90.5 ± 0.001
5	53.00 ± 0.23	63.6 ± 0.001
2.5	32.50 ± 0.15	36.3 ± 0.003
1.25	9.60 ± 0.39	9.63 ± 0.001
0.625	1.51 ± 0.74	2.32 ± 0.007

**Table 2:** Cytotoxic effect of Biobran on Vero E6.

Biobran Concentration (μg/mL)	Cytotoxicity (%)
100	91.91 ± 0.9
50	60.84 ± 2.3
25	38.99 ± 3.1
12.5	27.99 ± 0.2
6.25	24.8 ± 0.4
3.125	16.1 ± 0.3

**Table 3:** Antiviral activity of Biobran using in vitro Vero E6 toxicity and plaque reduction assays against SARS-CoV-2.

Biobran Concentration (μg/mL)	Initial Viral Count (PFU/mL)	Post Viral Count (PFU/mL)	Inhibition (%)
50	6.4 × 0^3^	0.6	90.6 ± 0.2
25	6.4 × 10^3^	1.6	75 ± 0.3
12.5	6.4 × 10^3^	2.9	55 ± 0.1
6.25	6.4 × 10^3^	4.7	27 ± 0.2

**Table 4:** IC_50_ and EC_50_ of Biobran.

Vero E6 IC_50_ (μg/mL)	Anti-COVID-19 EC_50_ (μg/mL)	EC_50_/IC_50_ (%)
15.36	3.46	22.5

**Table 5:** Effect of Biobran on SARS-CoV-2 gene expression and protein levels.

Biobran Concentration (μg/mL)	E-gene Expression	E-gene Down Regulation (%)	RdRp Expression	RdRp Down Regulation (%)	E-Protein Inhibition (%)	Spike Protein Inhibition (%)
50	0.073 ± 0.01	92.7 ± 1.2	0.07 ± 0.005	93 ± 2.8	91.3 ± 0.2	93.3 ± 0.4
25	0.266 ± 0.05	73.4 ± 1.9	0.19 ± 0.003	81 ± 3.1	75.3 ± 0.5	79.9 ± 0.7
12.5	0.44 ± 0.02	56.0 ± 0.9	0.36 ± 0.005	64 ± 1.5	49.6 ± 0.4	57.8 ± 0.7
6.25	0.78 ± 0.03	2 1± 1.2	0.68 ± 0.003	32 ± 0.9	19.4 ± 0.4	7.87 ± 0.2

## Data Availability

The data presented in this study are available on request from S.A. (for in vitro data) and from H.H.F. (for in silico data).

## Supplementary Materials

The following supporting information can be downloaded at: https://www.mdpi.com/article/10.3390/nu15020453/s1, Figure S1–S4. Figure S1. 3D image for position (model 0) of docking of RBD domain of SARS-CoV-2 spike protein (cyan color) to DC-SIGN CRD (light orange color) showing PPI via binding of V382 S383 T385 L387 N388 F392 F515 E516 L517 of chain A to L252 H254 P255 C256 P257 W258 of chain D, respectively, using cut-off 4.0 Å.; Figure S2. 3D image for position (model 1) of docking of RBD domain of SARS-CoV-2 spike protein (cyan color) to DC-SIGN CRD (light orange color) showing PPI via binding of E340 R346 A347 F348 N354 chain A to E259 of chain E and H254 and C384 of chain C, respectively, using cut-off 4.0 Å.; Figure S3. 3D image for position (model 2) of docking of RBD domain of SARS-CoV-2 spike protein (cyan color) to DC-SIGN CRD (light orange color) showing PPI via binding of N360 R466 D467 I468 S469 T470 I472 G482 V483 E484 T523 of chain A to N276 H278 D279 E286 of chain B and E286 V287 K379 A382 S383 C384 of chain C, respectively, using cut-off 4.0 Å.; Figure S4. 3D image for position (model 3) of docking of RBD domain of SARS-CoV-2 spike protein (cyan color) to DC-SIGN CRD (light orange color) showing PPI via binding of G381 V382 S383 P384 L387 N388 F392 T430 L517 A522 of chain A to L252 C253 H254 P255 P257 W258 E259 V287 A382 S383 C384 of chain D, respectively, using cut-off 4.0 Å.
