# Supplementary figures and images for: Biobran/MGN-3, an Arabinoxylan Rice Bran, Protects against Severe Acute Respiratory Syndrome Coronavirus 2 (SARS-CoV-2): An In Vitro and In Silico Study
Source: Nutrients. 2023 Jan 15;15(2):453. doi: 10.3390/nu15020453 (PMC9866808; doi:10.3390/nu15020453)

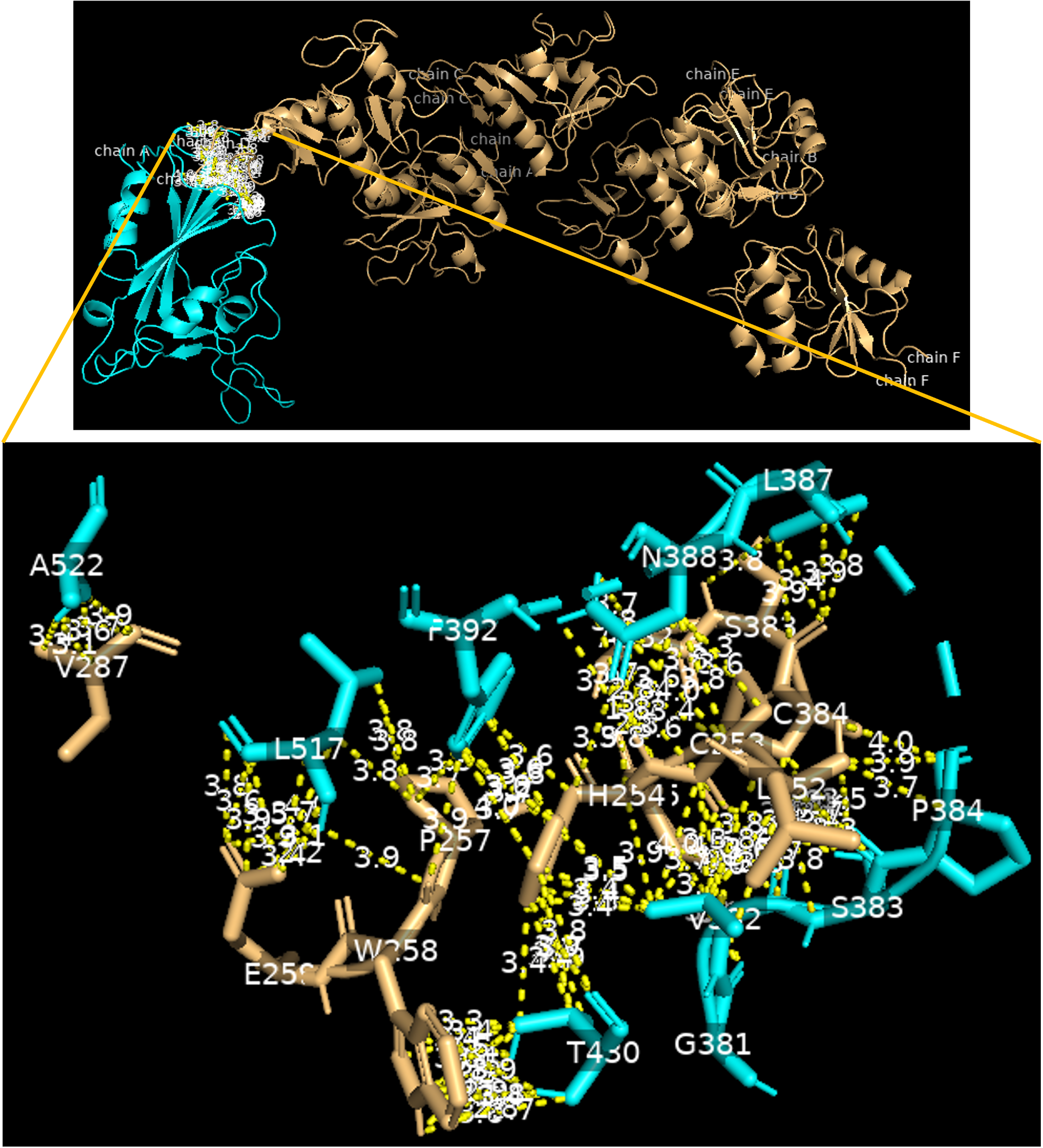

Supplement: Supplementary file 1 [file nutrients-15-00453-s001.zip › FigureS4.png]

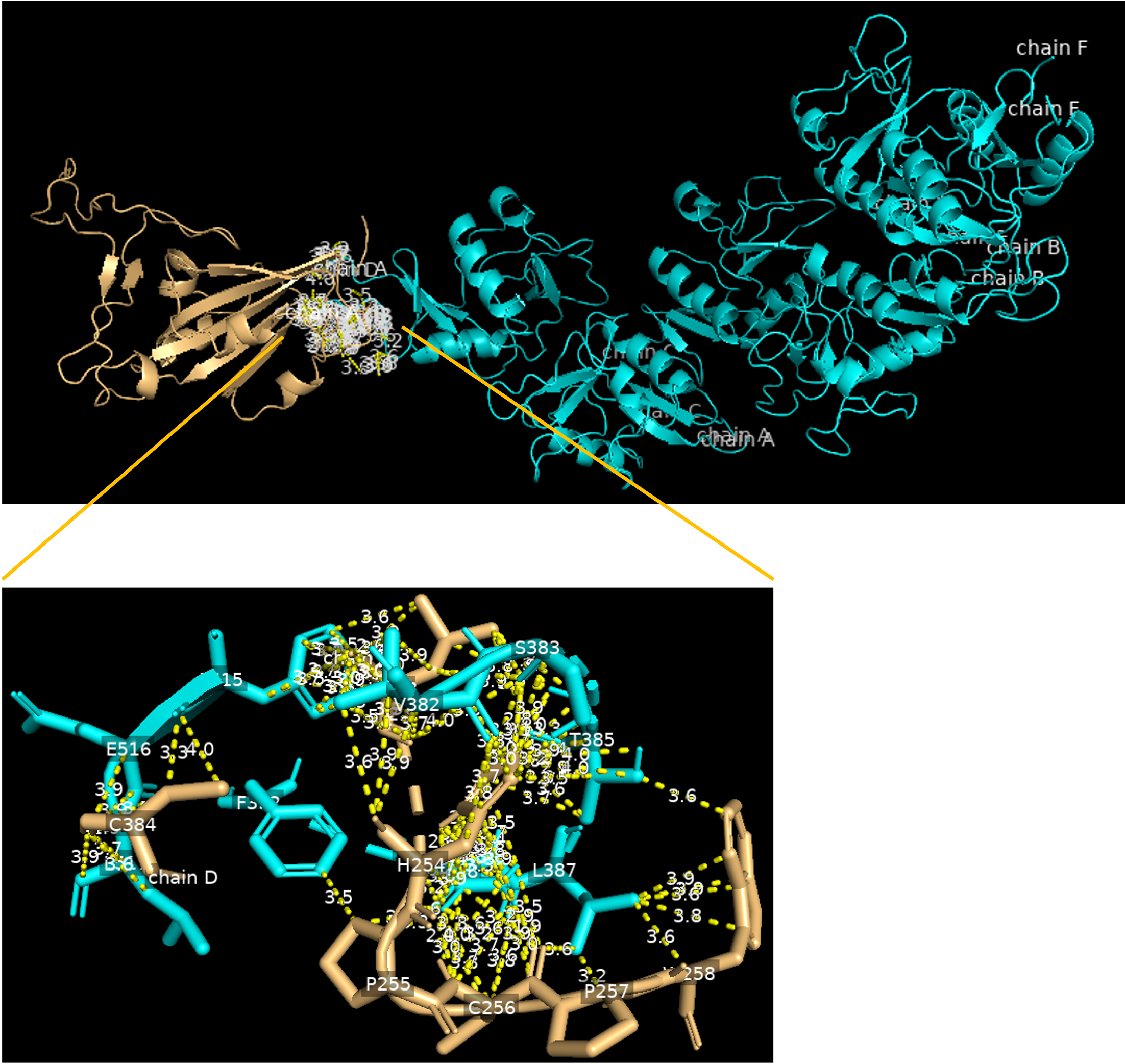

Supplement: Supplementary file 1 [file nutrients-15-00453-s001.zip › FigureS1.png]

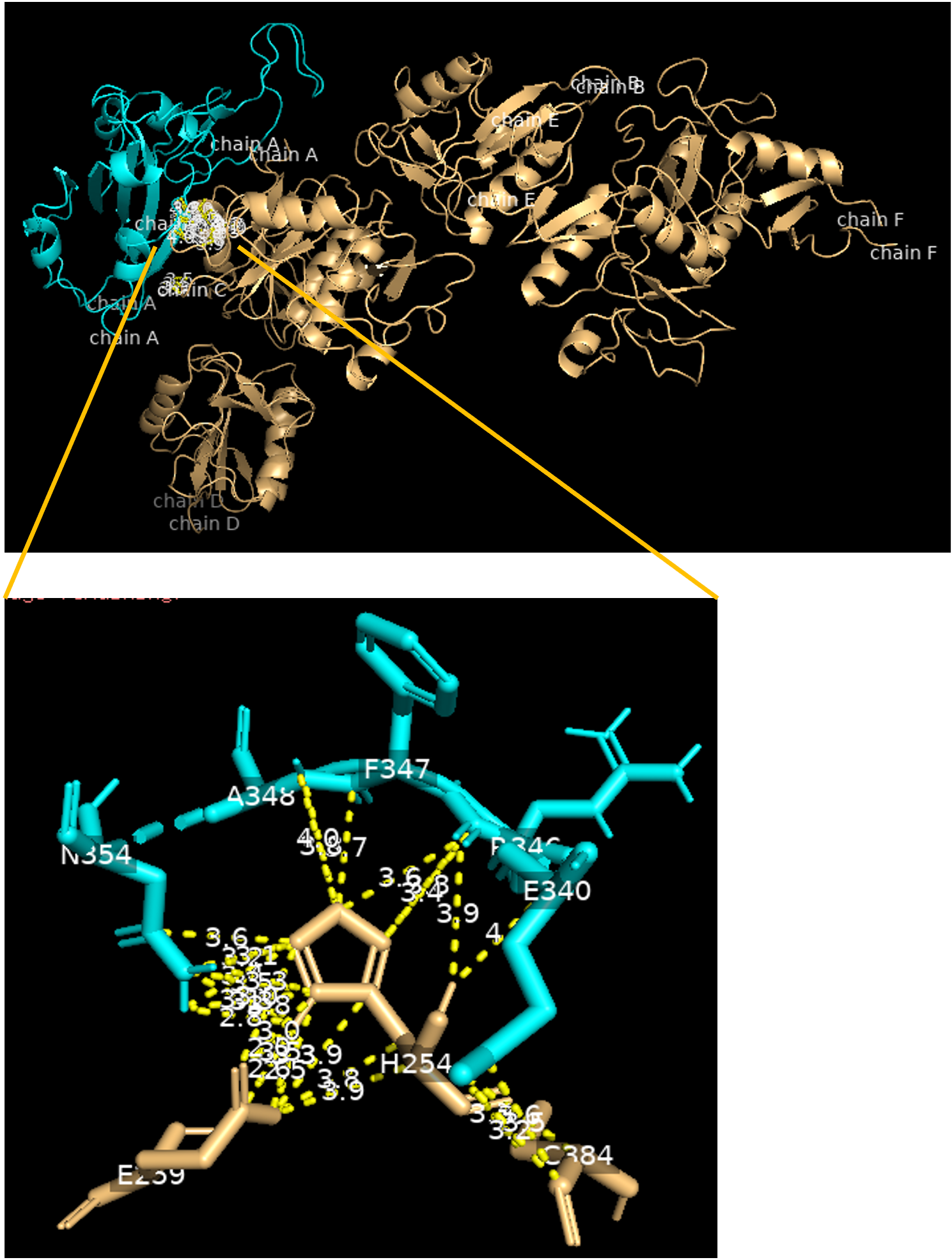

Supplement: Supplementary file 1 [file nutrients-15-00453-s001.zip › FigureS2.png]

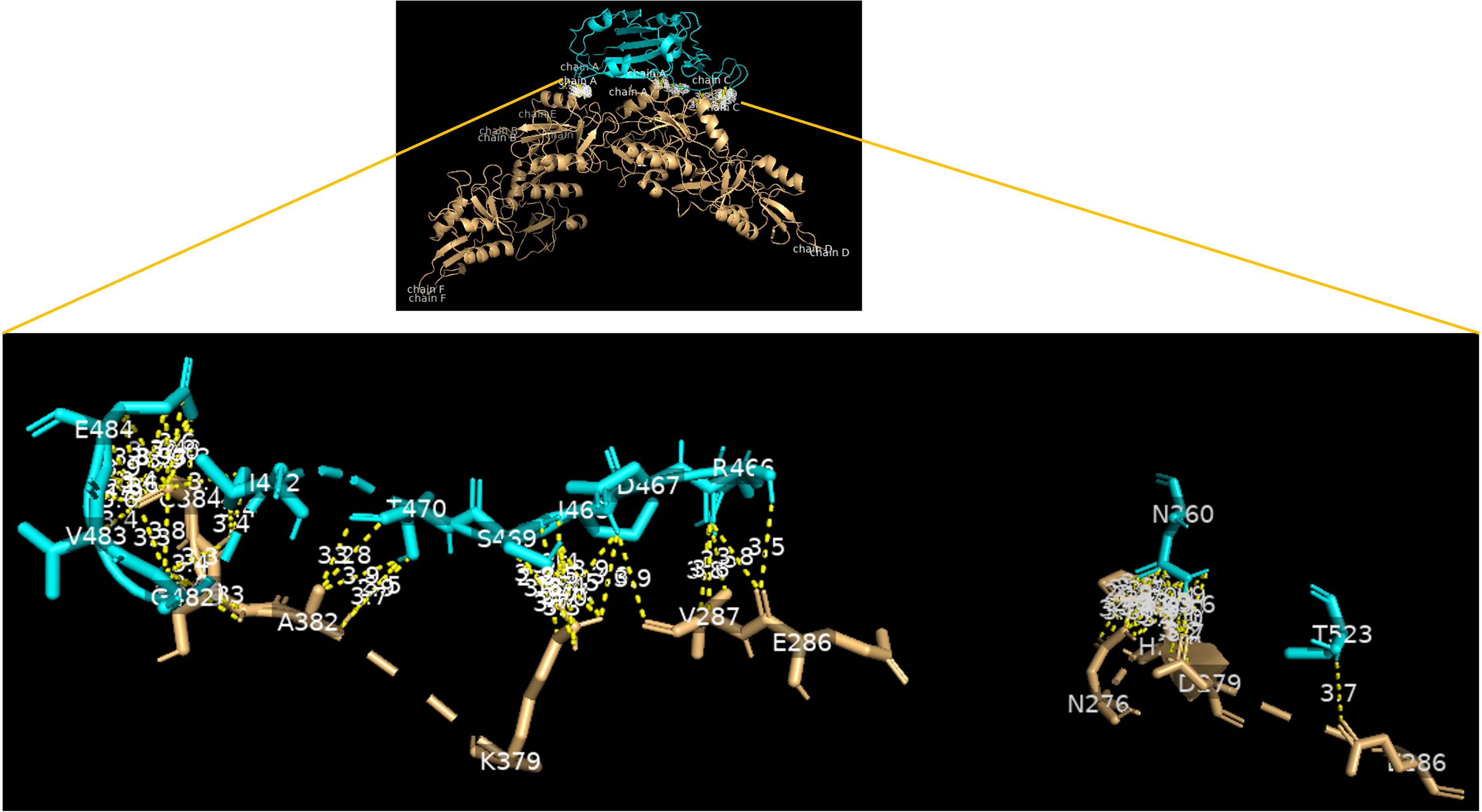

Supplement: Supplementary file 1 [file nutrients-15-00453-s001.zip › FigureS3.png]
